# Therapeutic challenges in central nervous system viral infections: advancing mesenchymal stem cell-based strategies for treating neuroinflammation and promoting tissue repair

**DOI:** 10.3389/fimmu.2025.1677433

**Published:** 2025-10-29

**Authors:** Javier Carbone-Schellman, Javiera Fontecilla-Escobar, Nicolás Sales-Salinas, William F. Chaparro-Pico, Alfredo Molina-Berríos, María Celeste Ruete, Pablo A. González, Mayra A. Machuca, Ma. Cecilia Opazo, Marcelo E. Ezquer, Luisa F. Duarte

**Affiliations:** ^1^ Centro de Medicina Regenerativa, Facultad de Medicina, Clínica Alemana – Universidad del Desarrollo, Santiago, Chile; ^2^ Millennium Institute on Immunology and Immunotherapy, Santiago, Chile; ^3^ Laboratorio de Cannabinoides y Biología Espermática, Instituto de Histología y Embriología de Mendoza, Consejo Nacional de Investigaciones Científicas y Técnicas, Universidad Nacional de Cuyo, Mendoza, Argentina; ^4^ Escuela de Microbiología, Universidad Industrial de Santander, Bucaramanga, Colombia; ^5^ Institute for Research in Dental Sciences, Faculty of Dentistry, Universidad de Chile, Santiago, Chile; ^6^ Facultad de Ciencias Biológicas, Pontificia Universidad Católica de Chile, Santiago, Chile; ^7^ Instituto de Ciencias Naturales, Facultad de Medicina Veterinaria y Agronomía, Universidad de Las Américas, Santiago, Chile; ^8^ Centro de Investigación en Ciencias Biológicas y Químicas, Universidad de Las Américas, Santiago, Chile

**Keywords:** virus, mesenchymal stem cells, central nervous system (CNS), neuroinflammation, secretome, extracellular vesicle (EV), neurological and neurodegenerative diseases, persistent infections

## Abstract

Although significant progress has been made in medicine and antimicrobial research, viral infections continue to pose a critical global health challenge, particularly when they involve the central nervous system (CNS). Despite advances in vaccines, antiviral agents, and small molecule therapeutics, current strategies remain insufficient to address the complex consequences of many CNS infections fully. Notably, many viruses are neurotropic and can invade the CNS, triggering infectious neuroinflammation that often lead to chronic neurological disorders and lasting morbidity. Current therapeutic approaches are largely ineffective in preventing or reversing this long-term neurological damage, underscoring the urgent need for innovative prophylactic and therapeutic interventions. Mesenchymal stem cells (MSCs) have emerged as a promising strategy to counteract chronic neuroinflammation and promote tissue repair following viral CNS infections. This review provides a comprehensive overview of CNS viral infection and neuroinflammation, including epidemiology and pathophysiology, and critically examines the limitations of existing treatments, particularly their inability to mitigate persistent neurological sequelae. Furthermore, we summarize recent preclinical and clinical studies investigating the use of MSCs in the context of CNS viral infections, highlighting their immunomodulatory and neuroprotective mechanisms, and discuss the challenges and future directions for MSC-based therapies in clinical settings.

## 1 Introduction

Neuroinfection is a worldwide concern and an important cause of morbidity and mortality, characterized by pathogen invasion of the central nervous system (CNS), including the brain and spinal cord, which can lead to severe neurological damage and long-term disabilities due to chronic neuroinflammation (1).

Neuroinflammation is a physiological response induced as a defense mechanism against microbial infections or CNS injury. While this process can exert a protective role that contributes to pathogen clearance and the reestablishment of tissue homeostasis, it can also become persistent and exacerbated, often resulting in irreversible neurological damage and long-term cognitive or motor deficits, hearing loss, seizures, coma, and even death (2).

The CNS is protected by specialized protective structures, including the blood-brain barrier (BBB) and the blood-cerebrospinal fluid barrier (BCSFB), as well as resident immune cells such as microglia and astrocytes, all of which function to limit pathogen entry and protect the neural tissue (3). However, several pathogens have evolved strategies to bypass these defenses and provoke diseases like encephalitis, meningitis, and demyelinating pathologies (4). The incidence of infectious neuroinflammatory diseases remains challenging to determine due to the heterogeneity of etiological agents, limitations on epidemiological surveillance, and underreporting, especially regarding neurological complications and long-term outcomes. Nevertheless, it was estimated by the 2019 Global Burden of Disease Study that the total number of meningitis cases in all age groups reached 2.52 million, with an estimated 236 thousand deaths (5). On the other hand, in 2019, a total of 1,444,720 cases of encephalitis were estimated. Importantly, while encephalitis and meningitis represent the most severe clinical manifestations of neuroinfection, a larger proportion of affected individuals have milder symptoms, especially in the post-acute phase or chronic phases of infection (6).

Current treatments primarily target the causative agent, aiming to control the infection and, in some cases, attenuate the associated neuroinflammation (7, 8). However, these strategies often fail to address the chronic consequences of neuroinflammation, such as persistent neurological and cognitive impairments (9). Due the threat of emerging pathogens, antimicrobial resistance, and the limitations of current therapies in limiting the lifelong sequelae, the search for new venues for treating neuroinfection constitutes a critical public health problem. In this context, mesenchymal stem cell (MSC)-based therapies have emerged as a novel and promising approach to modulate neuroinflammatory responses and promote CNS repair (10–12). Preclinical and clinical studies support their benefits across diverse conditions, including cardiac injuries, autoimmune and neurodegenerative diseases such as Parkinson’s, Alzheimer’s, Huntington’s, and Multiple Sclerosis (MS) diseases (13–16). These findings have encouraged further exploration of MSC-based therapies as a prospective candidate to treat the detrimental effects of infection-induced neuroinflammation (15).

Although a wide array of microorganisms, including viruses, bacteria, fungi, and parasites, can cause CNS infections, this review will focus on viral pathogens, particularly those with well-reported neurotropism and high prevalence, such as human herpesviruses (herpes simplex virus type 1 and Epstein Barr virus), human retroviruses (human immunodeficiency virus type 1 and the human T-lymphotropic virus type 1), severe acute respiratory syndrome coronavirus 2 (SARS-CoV-2), and Zika Virus (1). In the following sections, we summarize the current state of the art and critically examine the potential of MSC to address the unmet clinical needs in the treatment of virus-induced neuroinflammatory conditions.

## 2 CNS viral infections and current treatment challenges

Several viruses can invade the CNS, leading to acute infection causing encephalitis, meningitis, among other diseases, all commonly accompanied by neuroinflammation. Despite advances in antiviral therapies and supportive care, effective treatments remain limited, particularly for managing the immune-mediated damage and persistent neurological symptoms that often follow viral clearance. This section provides an overview of the most relevant viral pathogens associated with CNS infections and examines the limitations of current therapeutic approaches used to combat their associated diseases.

### 2.1 Herpes simplex virus type 1 (HSV-1)

HSV-1 is a highly prevalent neurotropic virus that belongs to the *Orthoherpesviridae* family. It establishes lifelong latency in sensory neurons and can reactivate under stress or immune suppression (17, 18). HSV-1 infection of the CNS may lead to severe acute encephalitis (herpes simplex encephalitis, HSE), with a high mortality rate in untreated patients and high morbidity with neurological sequelae in survivors treated with the currently available antivirals. Notably, asymptomatic brain infection has also been reported in an important proportion of healthy individuals (>35%) and it is associated with residual chronic neuroinflammatory responses that are believed to lead to neurodegeneration (19).

**Figure 1:**
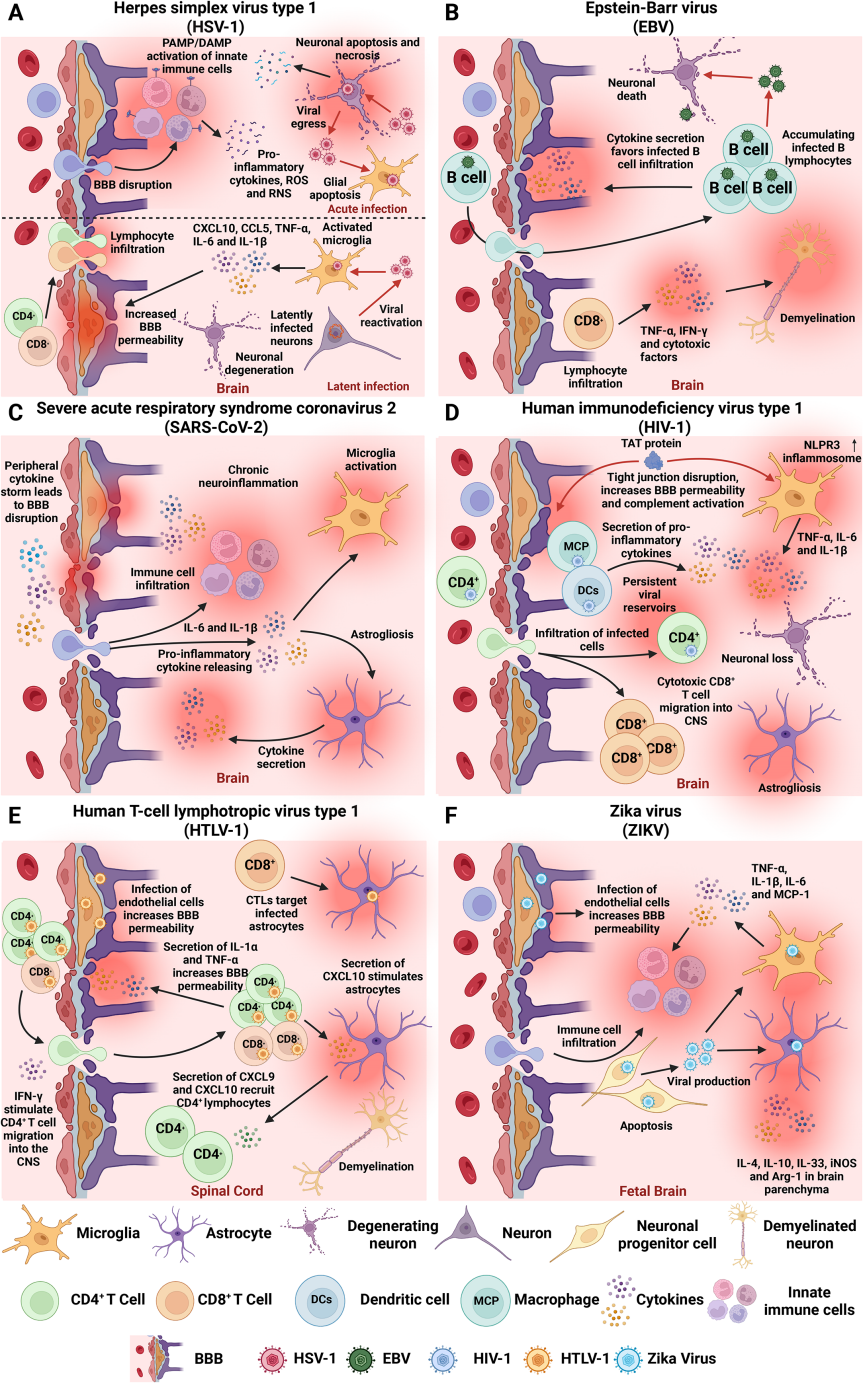
Neuroinflammation-associated hallmarks of viral infections in the CNS. Overview of key mechanisms by which prevalent human neurotropic viruses induce acute and/or chronic neuroinflammation, contributing to CNS dysfunction and long-term neuropathology. **(A)** HSV-1 can reach the CNS and cause an acute infection and herpes simplex encephalitis (HSE), characterized by neuronal and glial apoptosis and necrosis, BBB disruption, and massive innate immune cell infiltration. Latent infection is associated with sustained microglial activation and elevated levels of TNF-α, IL-6, IL-1β, CXCL10, and CCL5, promoting long-term neuroinflammation and tissue damage. **(B)** EBV establishes lifelong latency in B cells and has been associated with autoimmune and neurodegenerative disorders. The virus compromises BBB integrity, which facilitates CNS infiltration of infected B cells and triggers neuroinflammatory responses characterized by TNF-α and IFN-γ production, that can lead to chronic neuroinflammation and neurodegeneration. EBV-infected B cells and cytotoxic T cells accumulate in CNS tissues and can induce demyelination. **(C)** SARS-CoV-2 disrupts the BBB via systemic inflammation and cytokine storms. This leads to glial activation, increased IL-6 and IL-1β in CSF, and hippocampal injury. Other neuroinflammatory hallmarks include astrogliosis, microglial activation, and neuron damage. **(D)** HIV establishes reservoirs in CNS-resident macrophages and microglia early in infection. BBB disruption facilitates viral entry through a “Trojan Horse” manner into infected cells, enhanced by HIV Tat protein, which disrupts endothelial tight junctions and activates the complement cascade. Tat also triggers NLRP3 inflammasome activation and cytokine release by microglia. **(E)** HTLV-1 infects endothelial cells, promoting T cell infiltration and cytotoxic targeting of infected astrocytes. Infected immune cells infiltrate the spinal cord, where cytotoxic CD8^+^ T cells target HTLV-1-expressing cells and induce cytokine-mediated damage. **(F)** ZIKV virus crosses the placenta, infects neural progenitor cells, and induces apoptosis and impaired neurogenesis. ZIKV compromises BBB integrity and induces microglial activation with cytokine release. BBB, blood-brain barrier; CNS, central nervous system; IL, interleukin; IFN, interferon; TNF-α, tumor necrosis factor alpha.

During acute brain infection, neuronal and glial apoptosis and necrosis are observed, as well as BBB disruption, and exacerbated infiltration of innate immune cells into the CNS (20, 21). On the other hand, the long-term damages in the brain, are characterized by microglia activation, that lead to the release of pro-inflammatory cytokines such as tumor necrosis factor-alpha (TNF-α), interleukin (IL)-6, IL-1β, and chemokines C-X-C motif ligand (CXCL) 10 and C-C motif ligand (CCL) 5, that in turn recruit circulating lymphocytes to the CNS promoting chronic neuroinflammation (**Figure 1A**) (22, 23). This pro-inflammatory response has also been associated with tissue damage and neurodegeneration (24). Due to these characteristics, the contribution of HSV-associated neuroinflammation to the development of neurodegenerative disorders, such as MS, and Alzheimer’s disease, among others, has been widely considered (25–28).

Current antivirals like acyclovir (ACV) suppress viral replication but do not prevent long-term brain damage, and resistance, though rare, complicates treatment (29, 30). Unfortunately, nearly 50% of HSE patients treated with intravenous ACV show permanent sequelae after 1 year and 20% of mortality (29). Corticosteroids offer limited benefit due to immunosuppression risks (31, 32). Moreover, vaccine efforts remain unsuccessful (17, 33, 34), highlighting the urgent need for therapies that combine antiviral, immunomodulatory, and neuroprotective effects (35–37).

### 2.2 Epstein-Barr virus (EBV)

EBV, the gammaherpesvirus 4, causes infectious mononucleosis and is linked to carcinomas, lymphomas, and autoimmune diseases (38–41). After primary infection, it establishes lifelong latency in B cells, with periodic reactivation that can disrupt immune function (41, 42).

Importantly, EBV can invade the CNS, compromise the BBB integrity, and directly infect neurons leading to inflammation, tissue damage, and neurocognitive impairment (43, 44). It has been implicated in Alzheimer’s, Parkinson’s, and Multiple Sclerosis through mechanisms such as cytokine release, autoantibody cross-reactivity, and demyelination (**Figure 1B**) (45–54).

Despite growing evidence linking EBV to neurological disorders, its pathogenesis remains incompletely understood, and effective treatments are limited (55). Some compounds, such as cimetidine, have been used to treat chronic EBV reactivation and EBV associated carcinomas (56). Other molecules, including antiretrovirals, have shown anti-EBV activity and have been reported to induce long-term remission in some neurological disorders (57–61).

### 2.3 Severe acute respiratory syndorme coronavirus 2 (SARS-CoV-2)

SARS-CoV-2 is an RNA positive single-strand virus responsible for the COVID-19 pandemic, which has led to substantial global morbidity and mortality since its emergence in 2019 (62). It not only affects the respiratory system but also induces neurological damage. Some individuals develop lasting neurological symptoms, known as “Long COVID” or “Post-Acute Sequelae of SARS-CoV-2” (PASC) including cognitive deficits, delirium, and encephalopathy, that persist months after infection (63–65).

Neuropathological findings indicate that CNS injury is largely indirect, driven by peripheral cytokine storms that lead to hypoxia, BBB disruption, and microglial activation (**Figure 1C**) (66–69). Pro-inflammatory cytokines, such as IL-6 and IL-1β, have been detected in the cerebrospinal fluid (CSF) of patients with SARS-CoV-2 (70). Given the limited lymphatic drainage capability of the brain, these cytokines have been linked to neuropsychiatric symptoms (71). Thus, early treatment of the pro-inflammatory response against SARS-CoV-2 is crucial for reducing that symptoms and long-term sequelae, ultimately leading to the development of PASC (72).

Importantly, vaccination has reduced morbidity and mortality (73–79), and drugs such as remdesivir and tocilizumab can improve acute outcomes (80, 81), but no therapies currently prevent or treat PASC effectively (82), highlighting the need for new strategies targeting neuroinflammation and chronic sequelae.

### 2.4 Human immunodeficiency virus (HIV)

HIV is a single-strand RNA virus that establish latency in CD4^+^ T cells and reservoirs in macrophages and dendritic cells (DCs) leading to immune suppression and acquired immune deficiency syndrome (AIDS) (83). This loss of immune function increases susceptibility to opportunistic infections and malignancies (84). Besides its immunosuppressive effects, it has been described a spectrum of HIV-associated neurocognitive disorder (HAND), ranging from cognitive impairments to HIV-associated dementia, with up to 50% of patients affected (85).

Although HIV does not directly target the CNS, these neurological symptoms have been associated with virus infiltration through a “Trojan horse” manner due to the establishment of persistent reservoirs in perivascular macrophages and microglia (**Figure 1D**) (86). Moreover, although antiretroviral therapy (ART) can control HIV replication, the neurocognitive impairment and neuropathology persist in the CNS, as evidenced in post-mortem analysis of brain tissues with reported microglial activation, astrogliosis, neuronal loss, among other pathophysiological hallmarks of chronic neuroinflammation (87).

Notably, the viral trans-activator of transcription (Tat) protein facilitates CNS entry by disrupting endothelial tight junctions, activating complement, and recruiting monocytes (**Figure 1D**) (88). This, together with immune activation cause that macrophages release neurotoxins, cytokines, and metabolites, leading to brain damage (89, 90). Tat also activates the NLPR3 inflammasome in microglia, enhancing the release of TNF-α, IL-6 and IL-1β, thereby contributing to neuroinflammation (91, 92). Moreover, CD8^+^ infiltration into the brain can cause HIV CD8^+^ encephalitis (CD8E), a severe neurological manifestation which can lead to coma or even death if not treated properly (93).

While ART has significantly reduced mortality and incidence of HAND, milder cognitive symptoms persist in a large proportion of patients, indicating that ART alone is insufficient to fully prevent or reverse HIV-associated CNS injury (94–97), highlighting the need for therapies that target CNS inflammation and neuronal injury.

### 2.5 Human T-lymphotropic virus type 1 (HTLV-1)

HTLV-1, the first human retrovirus to be identified (98), affects between 5 and 10 million people worldwide (99) and is transmitted vertically, by non-protected sexual relationships, or through contamination with blood products (100–102).

In addition to its established role in adult T-cell leukemia (103), HTLV-1 is also responsible for HTLV-1-associated myelopathy/tropical spastic paraparesis (HAM/TSP), a severe chronic inflammatory disease that involves the dysfunction of the spinal cord characterized by paraparesis, neurogenic bladder, and sensory disturbance of the legs (104–106). While HTLV-1 can infect multiple nucleated cells, the majority of the viral burden (≈95%) resides in CD4^+^ T cells (107, 108). This virus spreads from cell to cell via direct contact through the virological synapse, biofilm-like structures and cellular conduits (109).

In HAM/TSP pathology, T cell activation facilitates the migration of both CD4^+^ and CD8^+^ T lymphocytes from the peripheral blood by crossing the BBB. Within the CNS, HTLV-1-specific CD8^+^ T cells target HTLV-1 antigen-expressing cells, primarily infected CD4^+^ T cells and possibly infected glial cells (**Figure 1E**) (108), that in turn triggers the release of neurotoxic cytokines, contributing to tissue damage and demyelination (110, 111). After months or even years, the inflammatory process ends and, macroscopically, one of the signs found is the loss of spinal cord volume (112).

While no treatment is recommended in asymptomatic patients, corticosteroids can provide temporary symptom relief in progressive disease forms (113). On the other hand, antiretrovirals like zidovudine and lamivudine show inconsistent efficacy (114–117). Initial studies with Zidovudine reported both no clinical effect and improvements in patients (118, 119). Moreover, studies using drugs such as Lamivudine, Tenofovir, and Zidovudine + Lamivudine have shown either a reduction in viral load over a short period (120), *in vitro* activity (121), or conversely no decrease in viral load and no improvement in clinical symptoms (122). These findings highlight the need for novel therapeutic approaches capable of modulating the immune response, halting neurodegeneration, and improving functional outcomes in patients with HAM/TSP.

### 2.6 Zika virus (ZIKV)

ZIKV is a mosquito-borne human pathogen (123). This virus belongs to the *Flaviviridae* family, as do viruses such as Dengue virus and Yellow fever virus (124). This virus was linked to the 2015–2016 outbreak of congenital microcephaly, with thousands of cases of birth defects reported across the Americas (125–127).

Regarding the tropism of ZIKV for the CNS, studies have shown that the virus crosses the placenta and infects neural progenitor cells (NPCs) leading to apoptosis, disrupted neurogenesis and neurodevelopmental anomalies in the fetus (**Figure 1F**) (128). In adults, the infection can lead to neurological complications, such as Guillain‐Barre Syndrome, encephalitis, meningitis, myelitis and seizures (129). Severe brain infections are marked by presence of pro- and anti-inflammatory mediators, including IL-4, IL-10, IL-33, iNOS, and arginase 1 in brain parenchyma, suggesting a complex immune environment (130). Of these molecules, IL-33 is linked to necroptosis processes and may play a key role in ZIKV-induced neuronal injury (131).

ZIKV can also infect brain endothelial cells and compromise BBB integrity, facilitating viral entry and immune cell infiltration (132). Infection triggers a neuroinflammatory response characterized by the upregulation of cytokines and chemokines such as IL-6, TNF-α, IL-1β, and MCP-1 in microglia, promoting leukocyte recruitment and amplifying tissue damage (**Figure 1F**) (133).

Despite efforts in vaccine and antiviral development, no specific therapies are currently available to prevent or reverse ZIKV-induced neuroinflammation and its consequences.

### 2.7 Other viruses associated with chronic neuroinflammation

Japanese encephalitis virus (JEV), a mosquito-borne flavivirus, causes about 68,000 cases annually in Asia with 20–30% mortality (134, 135). During acute infection, JEV infects innate immune cells such as fibroblast, Langerhans cells, and macrophages, among others, activating antiviral pathways and secreting pro-inflammatory cytokines and chemokines (136). Once systemic replication is established, JEV can cross the BBB, likely facilitated by cytokine-induced permeability, and reaches the CNS (137). Inside the brain, JEV targets glial cells including microglia, inducing a pro-inflammatory state that leads to neuronal death and encephalitis (138–142). Clinical follow-up studies show that nearly half of survivors suffer long-term neurological sequelae, including cognitive deficits (143, 144). Importantly, no effective treatment exists (145). In 2021, a review comparing treatments against JE showed that only one of the twelve reports analyzed showed statistically significant positive outcomes after treatment (146). Minocycline showed only modest benefits without improving mortality or neurological outcomes compared to the placebo group (147).

Measles is a highly contagious infectious disease caused by Morbillivirus hominis formerly known as Measles virus (MeV) (148). This virus belongs to the *Paramyxoviridae* family and causes a disease with severe symptoms and complications and, in some cases, can lead to death (149). According to WHO, in 2018, nearly 142.000 measles-related deaths were reported, primarily among children under five (150). Subacute sclerosing panencephalitis (SSPE) is a chronic, progressive, and typically fatal infection of the CNS, caused by the persistence of MeV in the brain. Current therapies, such as interferon-α plus inosiplex have shown the highest rates of disease stabilization or improvement (151). However, continued research is needed to identify more accessible and effective treatments. Vaccination is the most effective preventive measure, yet vaccine hesitancy and incomplete coverage continue to drive outbreaks and severe complications (152–154).

**Figure 2:**
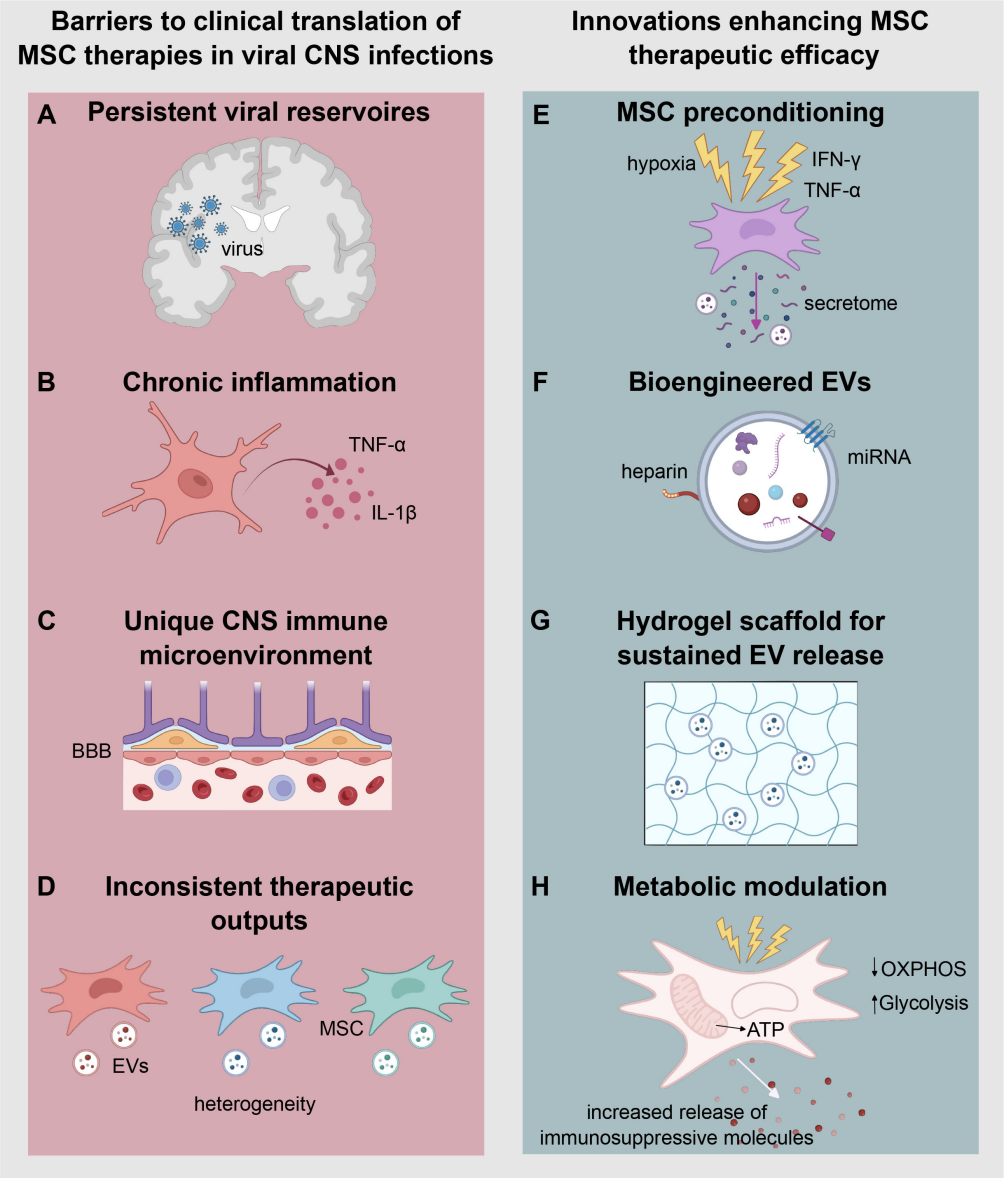
Barriers and emerging strategies for the clinical translation and enhancing therapeutic efficacy of mesenchymal stem cell therapies in viral central nervous system (CNS) infections. The left panel **(A–D)** illustrates key barriers to MSC-based therapeutic approaches in the context of viral CNS infections. **(A)** Persistent viral reservoirs promote chronic inflammation, which impairs the reparative function of MSCs. **(B)** Sustained production of pro-inflammatory cytokines such as TNF-α and IL-1β contributes to immune-mediated tissue damage. **(C)** The CNS immune microenvironment, characterized by the selective permeability of the blood-brain barrier (BBB), limits cell and therapeutic molecule access to the brain parenchyma. **(D)** MSC-based therapies are often hindered by inconsistent therapeutic outcomes due to donor heterogeneity and variability in the cargo of extracellular vesicles (EVs). The right panel **(E–H)** highlights emerging innovations aimed at overcoming these challenges and enhancing the therapeutic efficacy of MSCs. **(E)** MSC preconditioning using stimuli such as hypoxia, IFN-γ, or TNF-α enhances their anti-inflammatory and immunomodulatory secretome. **(F)** Bioengineered EVs, which incorporate targeted ligands and optimized miRNA content, improve brain targeting and functional delivery. **(G)** Hydrogels enable the sustained release of EVs, maintaining therapeutic concentrations in chronic inflammatory environments. **(H)** Metabolic modulation of MSCs promotes a shift from oxidative phosphorylation (OXPHOS) to Glycolysis, enhancing ATP production and driving the increased release of immunosuppressive molecules.

In summary, current treatment strategies to combat CNS injury by viral infections are often insufficient likely due to the poor penetration across BBB, which restricts therapeutic efficacy at the site of chronic damage (**Figures 2A-C**). Additionally, for many neurotropic viruses, vaccines are absent or insufficient to prevent neurological sequelae, highlighting the urgent need for novel therapies that combine antiviral efficacy with immunomodulation to prevent or mitigate long-term neuroinflammatory damage.

## 3 Mesenchymal stem cells as an alternative to treat neuroinflammation and promote CNS repair

MSC-based therapies, which include MSCs, extracellular vesicles (EVs), and their secretome, has demonstrated promising potential to reduce not only viral burden but also the associated neuroinflammatory response (14, 155, 156). In the next sections, we summarize the biological characteristics and immunomodulatory mechanisms of MSCs and their derivates, along with their applications in neuroinflammatory conditions.

### 3.1 Characteristics of mesenchymal stem cells and their secretome

MSCs are multipotent stromal cells with self-renewal capabilities and inherent tropism for injured tissues, including the inflamed CNS. According to the criteria established by the International Society for Cellular Therapy (ISCT), MSCs are characterized by adherence to plastic under standard culture conditions; expression of surface markers such as CD105, CD73, and CD90; and the absence of hematopoietic markers such as CD45, CD34, CD14, CD19, and HLA-DR (157, 158). Although MSCs can differentiate into osteoblasts, adipocytes, and chondroblasts *in vitro*, their therapeutic utility *in vivo* is primarily attributed to paracrine signaling rather than direct differentiation (159, 160).

MSCs are isolated from multiple tissue sources, each conferring distinct biological and clinical advantages. Bone marrow-derived MSCs (BM-MSCs) are the most extensively studied, with well-documented osteogenic and immunomodulatory properties; however, their isolation is invasive, and cell yield declines with donor age (161). Adipose-derived MSCs (AD-MSCs) are obtained through minimally invasive procedures, offering high cell yields and demonstrating therapeutic efficacy, with particular relevance in angiogenic applications (162). Umbilical cord-derived MSCs (UC-MSCs), particularly those from Wharton’s jelly, exhibit a primitive phenotype with enhanced proliferative capacity and lower immunogenicity, making them ideal for allogeneic transplantation (159). Emerging sources such as dental pulp, menstrual blood, and amniotic fluid are gaining attention due to their niche-specific advantages. For instance, dental pulp MSCs show strong neurogenic potential (163), while amniotic fluid-derived MSCs retain pluripotent-like properties (164).

The main therapeutic efficacy of MSCs is largely attributed to their secretome, which comprises a complex repertoire of growth factors such as brain-derived neurotrophic factor (BDNF) and vascular endothelial growth factor (VEGF), as well as immunoregulatory cytokines (e.g., IL-10, TGF-β), and EVs carrying regulatory miRNAs, mRNAs, and proteins that modulate immune and neuroinflammatory pathways which promote neuroprotection and tissue repair (165–167). EVs, especially exosomes, have garnered increasing interest by influencing cellular behavior without the risks associated with live cell therapies. EVs have shown anti-inflammatory and neuroprotective effects in various models of CNS diseases. Notably, EVs exhibit an enhanced capacity to cross the BBB compared to whole MSCs, thereby increasing their CNS bioavailability (168). Importantly, MSCs display a natural ability to migrate to sites of inflammation, a phenomenon guided by the expression of chemokine receptors such as CCR2, CXCR4, and CX3CR1 and their ligands secreted by inflamed tissues (169). These properties establish MSCs and their derivatives as strong candidates for cell-based therapies targeting the immune-mediated components of CNS viral infections.

### 3.2 Current applications of MSCs in neuroinflammation and CNS repair

MSCs secrete a diverse array of bioactive molecules, including cytokines, growth factors, and EVs loaded with regulatory microRNAs and proteins that collectively suppress neuroinflammation and promote tissue repair (155, 170–172). A key mediator, the tumor necrosis factor-inducible gene 6 protein (TSG-6), binds to CD44 receptors on microglia and macrophages, inhibiting TLR2/NF-κB signaling and reducing proinflammatory cytokines (e.g., TNF-α, IL-1β), and enhancing anti-inflammatory cytokines such as IL-10 and TGF-β (168, 173). This mechanism disrupts the feed-forward cycle of neuroinflammation, a hallmark in viral CNS complications, such as HAND and HSE (173). Secreted cytokines like IL-10, TGF-β, and PGE2 suppress pro-inflammatory Th1/Th17 responses and promote regulatory T cell expansion, which could attenuate cytokine-driven damage during viral CNS infections (174, 175), while neurotrophic factors such as BDNF, nerve growth factor (NGF), hepatocyte growth factor (HGF), and VEGF enhance neuronal survival and synaptic plasticity (176, 177).

Moreover, microRNAs-containing EVs (e.g., miR-134, miR-138-5p, miR-21-3p) reduce oxidative stress and neuronal apoptosis by modulating pathways such as the KDM6B–BMP2/BMF axis (178). These miRNAs have been shown to promote neuronal survival and functional recovery in experimental models of neuroinflammation (179, 180). Notably, miRNAs display greater stability and functionality when encapsulated in EVs, compared to the broader MSC secretome, where their half-life is significantly shorter due to enzymatic degradation (181).

Furthermore, MSCs and their EVs modulate the neuroimmune microenvironment by inducing microglial polarization from a proinflammatory M1 to an anti-inflammatory M2 phenotype (173). This phenotypic shift fosters a regenerative niche that supports synaptic repair and axonal regeneration. Regarding spinal cord injury models, MSC-EVs promote axonal regeneration by modulating the Rho-GTPase pathway, enhancing synaptic reconnection and functional recovery even during chronic stages (182, 183). Clinical trials in traumatic brain injury (TBI), have shown that intravenous MSC administration within 48 hours post-injury resulted in reduced plasma neurofilament light chain (NfL) levels, suggesting decreased axonal injury (184). The MATRIx phase II trial also showed greater white matter integrity and improved functional recovery with MSC therapy compared to placebo (185). In neuroinflammatory conditions such as MS and TBI, early-phase clinical trials have shown MSC infusions safety and some improvement in neurological function and reductions in systemic and CNS inflammation (186, 187).

Additionally, the MSC secretome plays a crucial role in modulating the neurogenic niche of the subventricular zone (SVZ), a key neurogenic area in the adult brain (188, 189). The SVZ harbors endogenous neural stem cells capable of proliferating, differentiating, and migrating to sites of injury or inflammation, thereby facilitating intrinsic brain repair (189–191). Factors secreted by MSCs and their EVs promote the proliferation and differentiation of these neural progenitor cells, enhancing neurogenesis and contributing to neuronal regeneration (189, 192). This paracrine effect complements the immunomodulatory functions of MSCs, expanding their therapeutic potential to repair neural damage in the context of chronic CNS inflammation, such as in viral infections (193).

## 4 Advancing MSC-based therapies from bench to bedside for treating CNS viral pathologies

Although MSCs are not inherently antiviral agents, these cells and their derivatives may trigger a host response that indirectly suppresses viral replication helping to preserve the integrity of the BBB and reducing inflammation (194). In preclinical models, MSC-based treatments have shown to reduce viral titers and improve clinical outcomes (155, 195, 196). Moreover, clinical investigations are underway to validate the safety, tolerability, and efficacy of MSC-based therapies in multiple indications, as well as for determining optimal doses and delivery strategies. However, studies specifically targeting viral infections in the CNS remain limited and face significant regulatory and scalability challenges, as discussed in the following sections (177).

### 4.1 Preclinical evidence supporting the use of MSC-based therapies in viral infections and neuroinflammatory conditions

MSC co-cultured with JEV-infected microglia and neurons *in vitro* have shown the capacity to promote microglial polarization towards an anti-inflammatory M2 phenotype, enhance neuronal survival, and reduce viral replication (155). Consistent with this, mice infected with JEV and treated with BM-MSCs displayed a reduced mortality, neuroinflammation, and viral load, accompanied by decreased microglial activation, lower levels of pro-inflammatory cytokines such as TNF-α, IFN-γ, and CCL-2, preservation of the BBB, and increased expression of type I interferons (IFN-α/β) (155).

Similar findings have been reported for coxsackievirus B3 (CVB3)-induced myocarditis. Human BM-MSCs reduced apoptosis, oxidative stress, and viral replication, through mechanisms dependent on nitric oxide production induced by IFN-γ priming (196). Moreover, exosomes derived from human UC-MSCs alleviated inflammation and apoptosis in CVB3-infected human cardiomyocytes through activation of the AMPK/mTOR autophagy pathway, promoting cell survival and protein degradation (197). In murine models, intravenous administration of BM-MSCs or their EVs improved cardiac function, decreased myocardial apoptosis and inflammation, and lowered TNF-α (196–198).

Promising results also emerge in herpesviruses infections. In CMV-infected murine macrophages, exosomes from BM-MSCs shifted macrophage phenotype from pro-inflammatory M1 to anti-inflammatory M2 phenotypes and reduced pro-inflammatory cytokines (199). In murine models of CMV-induced pneumonia, MSC-exosomes significantly improved survival, and reduced weight loss, lung damage, inflammatory cell infiltration, and pulmonary fibrosis. These effects were associated with reduced NF-κB activation and suppression of the NLRP3 inflammasome (199). Moreover, conditioned media derived from bone marrow–isolated MSCs in mice has been shown to inhibit HSV-1 infection *in vitro*. Furthermore, administration of BM-MSCs in a lethal HSV-1 mouse model conferred 70% protection, compared with only 10% survival in untreated animals. MSC treatment was also associated with enhanced production of virus-neutralizing anti-HSV-1 antibodies and increased T-cell proliferation (200). Another study demonstrated that prior immunization with MSCs protected all mice from lethal HSV-1 infection, while genetically modified MSCs transfected with the *Us6* gene encoding glycoprotein D conferred 65% protection compared to untreated mice that had 100% mortality (201). These findings highlight the unique capacity of MSCs to stimulate innate, adaptive, and protective immunity representing promising candidates for the development of next-generation cell-based vaccines against herpes and other viral infections.

Although CMV, HSV, and VZV can infect MSCs *in vitro*, viral replication is limited, possibly associated with constitutive expression of interferon-stimulated genes (ISG) (202, 203). In the same line, MSCs are non-permissive to EBV and HHV-6/7 infections (204). However, the direct antiviral or immunomodulatory effects of MSCs, their exosomes, or their secretome in the context of VZV, EBV, or HHV-6/7 infections remain largely unexplored. Further studies are needed to define the therapeutic potential of MSC-based interventions on the diseases caused by these viruses.

Notably, EVs derived from human Wharton’s Jelly MSCs, showed a dose-dependent inhibition of HSV-2 in Vero cells assays (205). At non-toxic doses, EVs reduced viral spread and cytopathic effects (205). Their antiviral mechanism may involve direct virucidal activity and interference with viral replication, possibly via antiviral microRNAs (205).

In HIV models, MSC therapy showed a dual effect. When were used on two latently infected U1 (monocytic) and ACH2 (T-cell) lines, MSCs could reactive latent virus via the PI3K and NF-κB pathways but also enhanced the efficacy of latency-reversing agents (LRAs) like panobinostat and Phorbol 12-myristate 13-acetate (PMA) (206). MSCs also demonstrated *in vivo* potential to restore immune balance, reduce neuroinflammation and modulate microglial activation, with possible benefits in HAND (207).

MSC-based therapies have been explored to mitigate cytokine storms and organ damage after SARS-CoV-2 infection. In preclinical models of acute respiratory distress syndrome (ARDS), MSCs improved oxygenation, reduced alveolar inflammation, and preserved endothelial integrity (208–210). MSC-derived EVs have also been proposed to treat COVID-19-associated neuroinflammation and stroke, through immune modulation and neuro-regenerative effects (211).

MSC and their secretome modulate multiple arms of antiviral immunity mechanisms that could in principle facilitate the viral clearance and disease resolution during acute infections. Indeed, they can produce type I interferons and secrete chemokines, that recruit and activate plasmacytoid, myeloid dendritic cells, and enhance NK and CD8^+^ T cell functions (212, 213). Once the infection has been resolved, MSCs shift toward anti-inflammatory phenotypes, releasing mediators such as TGF-β, indolamine 2,3-dioxygenase (IDO), IL-10, IL-1Ra, and PGE2, which control excessive immune activation and prevent cytokine storms (194, 203, 214). However, certain viruses, including HIV, HSV and hepatitis B virus (HBV), can infect MSC and potentially establish viral reservoirs, raising safety concerns. Therefore, donor MSCs must be rigorously screened for these pathogens prior to transplantation to minimize the risk of virus-associated complications and ensure the safety of MSC-based therapies.

In summary, while MSC show promising effects in acute viral infection by reducing viral load and dampening acute inflammation, evidence for their role in clearing persistent or latent infections is weaker. MSCs have been shown to reactivate latent HIV-1 in monocytic and T-cell models through activation of a noncanonical PI3K–NFκB–dependent pathway (206). Although this reactivation may represent a therapeutic leverage point, whether it leads to immune-mediated clearance *in vivo* remains to be determined. In persistent infections, MSCs could potentially serve as adjuvants to conventional antiviral therapy. Supporting this, exosomes derived from UC-MSCs have been reported to enhance the antiviral effects of interferon-α and telaprevir against hepatitis C *in vitro*, suggesting additive or even synergistic activity; these exosomes carry microRNAs that directly complement viral genomes within host cells (215).

### 4.2 Limited clinical evidence supporting MSC-based therapies for viral CNS infections

While preclinical studies suggest that MSCs can attenuate neuroinflammation, protect neurons, and indirectly suppress viral replication, clinical evidence in humans, particularly in cases of viral encephalitis and other CNS viral infections remains limited (216, 217). To date, most research has relied on rodent models or *in vitro* systems. Extrapolating these results to human applications requires caution, given substantial interspecies differences in immune response dynamics and CNS architecture (218).

**Figure 3:**
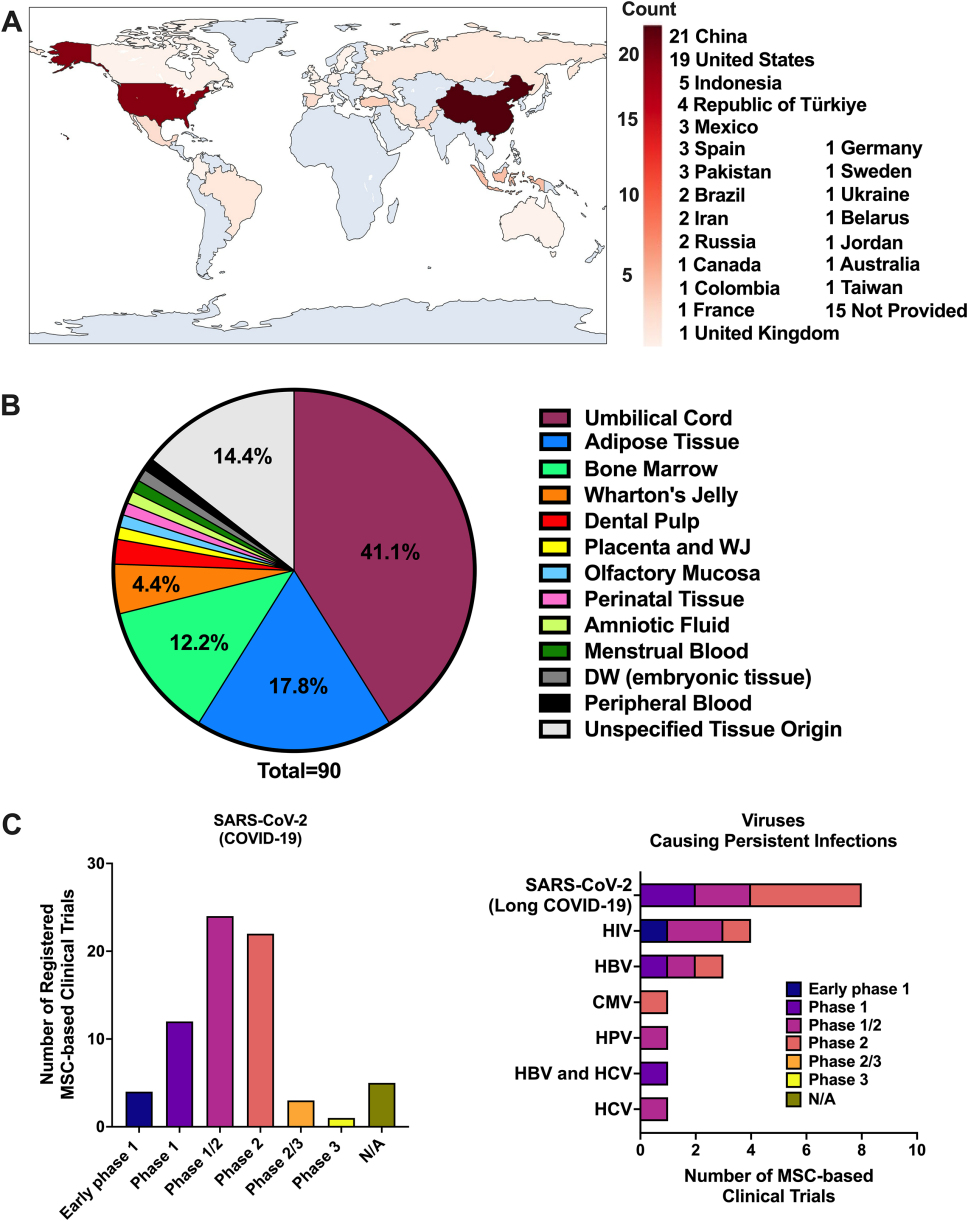
Summary of clinical trials on MSC-based therapies targeting viral infections. Overview of MSC-based clinical trials for viral infections. **(A)** Distribution of trials by country. **(B)** MSC sources used across studies. **(C)** Targeted viral pathogens, subdivided into SARS-CoV-2 acute infection/COVID-19 (left) and viruses associated with persistent infections, including long COVID due to SARS-CoV-2 (right). Data were obtained from the NIH ClinicalTrials.gov database by searching “virus” and “mesenchymal stem cells.” Of 107 identified studies, 90 were included after excluding those not using MSCs for viral infection treatment or targeting multiple viruses.

Human clinical studies with MSCs have mostly focused on systemic viral infections, especially COVID-19, where MSCs are used for immunomodulation rather than direct viral clearance (186, 219). A search of the National Institutes of Health database (http://www.clinicaltrials.gov/), using “virus” as the condition/disease and “mesenchymal stem cells” as other terms, identified 107 MSC-based clinical trials. After excluding studies that did not involve MSCs for the treatment of viral infections and those targeting more than one virus, a total of 90 trials were tabulated in **Figure 3**. The distribution of studies by country revealed a predominance in China and the United States (**Figure 3A**). UC-MSC, BM-MSC and AD-MSC were the most frequently utilized cell sources (**Figure 3B**). The majority of trials conducted to date are phase 1 and phase 2 studies designed to assess safety and feasibility, with limited evidence available so far regarding therapeutic efficacy (**Figure 3C**). Numerous MSC-based studies have been registered for treatment of COVID-19 related pneumonia and acute respiratory distress syndrome (ARDS). **Table 1** summarizes completed clinical studies involving MSC treatment for treating SARS-CoV-2 acute infections. MSC infusions demonstrated favorable safety and improved inflammatory profiles, supporting a substantial immunomodulatory potential in systemic viral infections that impact the CNS (238, 239).

**Table 1:** Completed clinical studies on clinicaltrials.gov evaluating the use of MSC or their derivates on SARS-CoV-2 infection.

Title of the trial	MSC source	Administration route	Primary outcome measure	Clinical findings	References/ID
Treatment With Human Umbilical Cord-derived Mesenchymal Stem Cells for Severe Corona Virus Disease 2019 (COVID-19)	hUC-MSCs	IV infusion	Evaluation of pneumonia Improvement; Change in lesion proportion (%) of full lung volume from baseline to day 28.	UC-MSC administration achieved a long-term benefit in the recovery of lung lesions and symptoms in COVID-19 patients after 1-year follow-up. Long-term safety was observed for the COVID-19 patients who received MSC treatment. However, efficacy of MSC treatment was not significantly sustained through the end of the 2-year follow-up period. Long-term safety of MSC therapy in patients with severe COVID-19 was observed over 3 years.	(220–223) https://clinicaltrials.gov/study/NCT04288102
Mesenchymal Stem Cells Therapy in Patients With COVID-19 Pneumonia	hUC-MSCs	IV infusion	Change in clinical symptoms, inflammatory markers, lung function, and oxygen requirement.	There was no statistically significant difference in terms of exit/discharge days and mortality between the groups: intubated with comorbidity (n:7), intubated without comorbidity (n:7) and no intubated (n:7). MSCs therapy showed reversal of hypoxia and downregulation of cytokine storm in patients with severe COVID-19.	(224) https://clinicaltrials.gov/study/NCT04713878
Clinical Use of Stem Cells for the Treatment of Covid-19	hUC-MSCs	IV infusion	Clinical symptoms improvement related to Covid-19 infection (fever, pneumonia, shortness of breath)	MSC transplantation seems to bring the cytokine storm under control and attenuate disease progression. MSC accelerated the recovery of damaged organs showing reduced mortality and decreased ICU stay.	(225) https://clinicaltrials.gov/study/NCT04392778
Efficacy of Intravenous Infusions of Stem Cells in the Treatment of COVID-19 Patients	hUC-MSCs	IV infusion	Safety and efficacy assessment of infusion associated adverse events and assessment of pneumonia improvement.	No published results	https://clinicaltrials.gov/study/NCT04437823
Mesenchymal Stem Cells for the Treatment of COVID-19	hUC- derived MSCs (PrimePro™)	IV infusion	Survival of hospitalized COVID-19 patients and infection rates in healthcare workers exposed to them.	Six out of 13 patients in the placebo group died during follow-up (46.15%), one out of 13 patients died in the treatment group (7.69%). No therapy-related side effects were documented in either group. No prophylactic effects were observed compared to placebo, as none of the healthcare workers developed COVID symptoms or tested positive at the end of the trial.	(226) https://clinicaltrials.gov/study/NCT04573270
Therapeutic Study to Evaluate the Safety and Efficacy of DW-MSC in COVID-19 Patients	DW-MSCs	IV infusion	Incidence of treatment-emergent adverse event in treatment group	Safe and well tolerated in low-risk COVID-19 patients; no disease progression; low treatment-emergent adverse events and no severe cases reported or related to the therapy.	(227) https://clinicaltrials.gov/study/NCT04535856
NestaCell^®^ Mesenchymal Stem Cell to Treat Patients with Severe COVID-19 Pneumonia	hIDPSC	IV infusion	Change in the clinical condition by ordinal scale (ordinal WHO scale that measures the severity of the disease over time)	NestaCell^®^ product is safe even for patients with increased risk for thromboembolism.	(228) https://clinicaltrials.gov/study/NCT04315987
Use of UC-MSCs for COVID-19 Patients	hUC-MSCs	IV infusion	Incidence, severity, timing, and treatment-relatedness of adverse and serious adverse events	Treatment was well tolerated, with significantly improved survival and patient recovery time was shorter, along with a significant reduction in adverse severe events, treatment related, with no serious infusion-related adverse events shortly after treatment.	(229) https://clinicaltrials.gov/study/NCT04355728
Mesenchymal Stem Cell Secretome in Severe Cases of COVID-19	hUC-MSCs Secretome	IV infusion	Assessment of inflammation marker levels	Safe and well tolerated, with no adverse events reported. Although no significant differences were observed in clinical symptoms, laboratory values showed the treatment effectively prevented the rise of inflammatory markers.	(230) https://clinicaltrials.gov/study/NCT05122234
Treatment of Severe COVID-19 Pneumonia with Allogeneic Mesenchymal Stem Cells	BM-MSCs (MSV^®^-allo)	IV infusion	Therapy success: IMV removal, and overall survival	No published results	https://clinicaltrials.gov/study/NCT04361942
Umbilical Cord Lining Stem Cells (ULSC) in Patients With COVID-19 ARDS	Umbilical Cord Lining Stem Cells	IV infusion	Incidence of dose-limiting toxicity, suspected adverse reactions, and treatment-emergent adverse and serious adverse events	No published results	https://clinicaltrials.gov/study/NCT04494386
An Exploratory Study of ADR-001 in Patients with Severe Pneumonia Caused by SARS-CoV-2 Infection	AD-MSCs	IV infusion	Safety: Adverse events which appear in subjects’ treatment.	No published results	https://clinicaltrials.gov/study/NCT04522986
A Proof-of-Concept Study for the DNA Repair Driven by the Mesenchymal Stem Cells in Critical COVID-19 Patients	hUC-MSCs	IV infusion	Gene expression of DNA repair-related genes examined in 5 different parts: to base excision (PARP1), nucleotide excision (NER), homologous recombinational (HR), and mismatch repair (MMR).	MSCs application had a significant effect on 6 genes located in 3 different DNA damage response pathways. NER pathway genes; RAD23 and ERCC1, HR pathway genes; ATM and RAD51, MMR pathway genes; MSH2 and MSH6.	(231) https://clinicaltrials.gov/study/NCT04898088
Treatment of Covid-19 Associated Pneumonia with Allogenic Pooled Olfactory Mucosa-derived Mesenchymal Stem Cells	Allogenic pooled olfactory mucosa-derived MSCs	IV infusion	Number of cured patients, assessed by PCR in addition to chest CT scan	Good tolerability and safety. In the study group, 43% of patients survived (6 out of 14), which was significantly higher than in the control group (5.6%, 1 out of 18). A correlation was found between lactate dehydrogenase (LDH) levels and the outcome of a severe form of coronavirus infection. Administration of pooled MSCs at LDH level higher than 519.8 U/l was ineffective.	(232) https://clinicaltrials.gov/study/NCT04382547
Menstrual Blood Stem Cells in Severe Covid-19	Allogeneic human menstrual blood stem cells secretome	IV infusion	Adverse reactions incidence and time to clinical improvement.	Well tolerated and safe; improved survival, lower need for intubation, reduced inflammation markers, increased CD4+/CD8+ cells, and better lung recovery.	(233) https://clinicaltrials.gov/study/NCT05019287
Cellular Immuno-Therapy for COVID-19 acute respiratory distress syndrome (CIRCA-19)	hUC-MSCs	IV infusion	Phase 1 trial: Determine safety of UC-MSCs in COVID-19 ARDS patients (using maximum feasible tolerated dose). Phase 2a trial: Early signs of benefit on morbidity and mortality. Ventilator-free days in patients with COVID-19 ARDS.	MSCs resulted in a non-statistically significant improvement in oxygen free-days, ICU-free days and survival, associated with a significant increase in lymphocyte count.	(234, 235) https://clinicaltrials.gov/study/NCT04400032
A Study of ADR-001 in Patients with Severe Pneumonia Caused by SARS-CoV-2 Infection (COVID-19)	AD-MSCs	IV infusion	Ventilator free days which appear in subjects with treatment.	No published results	https://clinicaltrials.gov/study/NCT04888949
Mesenchymal Stromal Cells for the Treatment of SARS-CoV-2 Induced Acute Respiratory Failure (COVID-19 Disease)	BM-MSCs	IV infusion	Proportion of participants with treatment-related serious adverse events and number of participants with improvement by at least two categories on a six-category ordinal scale at day 14.	Results posted on clinicaltrials.gov site. MSC therapy was safe, but the randomized results did not demonstrate clinical benefit over standard care and even suggested higher mortality in the MSC group.	https://clinicaltrials.gov/study/NCT04345601
Efficacy and Safety Study of Allogeneic HB-adMSCs for the Treatment of COVID-19	AD-MSCs	IV infusion	Analysis of inflammatory markers and incidence of adverse and serious adverse events (AE/SAEs).	Analyses of inflammatory markers showed increased levels of interleukin-6 and C-reactive protein over time in HB-adMSC group. Multiple infusions of 100MM allogeneic HB-adMSCs were considered safe for the study population.	(236) https://clinicaltrials.gov/study/NCT04362189
A Randomized, Double-Blind, Single Center, Efficacy and Safety Study of Allogeneic HB-adMSCs Against COVID-19.	AD-MSCs	IV infusion	Number of participants who were hospitalized due to symptoms by or associated with COVID-19.	Results posted on clinicaltrials.gov site. No participants receiving five IV infusions of HB-adMSCs were hospitalized or developed COVID-19 symptoms.	https://clinicaltrials.gov/study/NCT04348435
Multiple Dosing of Mesenchymal Stromal Cells in Patients with ARDS (COVID-19)	MSCs	IV infusion	Incidence of grade 3–5 infusional toxicities and predefined hemodynamic or respiratory adverse events related to the infusion of MSC.	Safety outcomes showed that zero patients experienced Grade 3–5 infusion-related toxicities or predefined hemodynamic or respiratory adverse events related to the infusion.	https://clinicaltrials.gov/study/NCT04466098
A Pilot Clinical Study on Inhalation of Mesenchymal Stem Cells Exosomes Treating Severe Novel Coronavirus Pneumonia	AD-MSCs Exosomes	Aerosol inhalation	Adverse reaction and severe adverse reaction due to treatment and time to clinical improvement.	No adverse or severe reactions observed; treatment well tolerated. clinical improvement in lung lesions, inflammation, and symptoms correlated with safety profile.	(237) https://clinicaltrials.gov/study/NCT04276987
The MEseNchymal coviD-19 Trial: MSCs in Adults with Respiratory Failure Due to COVID-19 or Another Underlying Cause	iPSC−MSC	IV infusion	Trend in trajectory of PaO2/FiO2 ratio between groups for the assessment of respiratory dysfunction.	No published results	NCT04537351 https://clinicaltrials.gov/study/NCT04537351
Efficacy of Infusions of MSC From Wharton Jelly in the SARS-Cov-2 (COVID-19) Related Acute Respiratory Distress Syndrome	WJ-MSCs	IV infusion	PaO2/FiO2 ratio for the assessment of respiratory dysfunction.	WJ-MSC treatment was safe and feasible in moderate/severe ARDS due to COVID-19 but did not improve PaO2/FiO2 >200 at day 10; primary endpoint not achieved.	(238) https://clinicaltrials.gov/study/NCT04625738
Cell Therapy Using Umbilical Cord-derived Mesenchymal Stromal Cells in SARS-CoV-2-related ARDS	Umbilical cord WJ-MSCs	IV infusion	Respiratory efficacy evaluated by the increase in PaO2/FiO2 ratio	No significant difference in PaO2/FiO2 change between UC-MSC and placebo groups; primary endpoint not met. A numerical improvement in the UC-MSC group suggests potential benefit warranting further study	(239) https://clinicaltrials.gov/study/NCT04333368
Investigational Treatments for COVID-19 in Tertiary Care Hospital of Pakistan	BM-MSCs	IV infusion	survival: death or recovery	No published results	https://clinicaltrials.gov/study/NCT04492501
Efficacy and Safety Evaluation of Mesenchymal Stem Cells for the Treatment of Patients with Respiratory Distress Due to COVID-19	WJ-MSCs	IV infusion	All-cause mortality: Number of patients who died, by treatment group	No published results	https://clinicaltrials.gov/study/NCT04390139
Extracellular Vesicle Infusion Treatment for COVID-19 Associated ARDS	BM-MSCs extracellular vesicles (ExoFlo)	IV infusion	Evaluation of 60-day Mortality Rate	ExoFlo showed a lower 60-day mortality in ARDS patients; not statistically significant in the intention-to-treat group, but significant reduction seen in 18–65 age subgroup. Suggests potential benefit, warrants larger trials.	(240) https://clinicaltrials.gov/study/NCT04493242
Intermediate Size Expanded Access Protocol for the Treatment of Post-COVID-19 Syndrome	AD-MSCs	IV infusion	Assessment of changes in neurological symptoms, vital signs, lab values, and physical exam findings.	Treatment with autologous HB-adMSCs resulted in significant improvements in the signs and symptoms associated with post-COVID-19 syndrome as assessed by visual analog scale and fatigue assessment scores.	(241) https://clinicaltrials.gov/study/NCT04798066
A First-In-Human Phase 1b Study of AmnioPul-02 in COVID-19/Other LRTI	MSC derived from amniotic fluid (AmnioPul-02)	IV infusion	Dose-limiting adverse events/toxicities	No published results	https://clinicaltrials.gov/study/NCT05348772
Evaluation of Safety and Efficiency of Method of Exosome Inhalation in SARS-CoV-2 Associated Pneumonia (COVID-19EXO).	MSC−Exosomes	Aerosol inhalation	Safety assessment: number of participants with non-serious and serious adverse events during trial and inhalation procedure.	Treatment safe and feasible; no serious adverse events related to the intervention or inhalation were reported in either group. Mild, transient adverse events occurred at similar rates in both groups, with no significant differences.	(242) https://clinicaltrials.gov/study/NCT04491240

hUC-MSCs, Human umbilical cord-derived MSCs; DW-MSCs, Human allogenic Daewoong Pharmaceutical’s MSCs; hIDPSC, Human immature dental pulp stromal cells; AD-MSCs, Adipose-derived mesenchymal stem cells; BM-MSCs, Bone Marrow Mesenchymal Stromal Cells; iPSC−MSC, mesenchymal stem cells derived from induced pluripotent stem cells; WJ-MSCs, Mesenchymal Stem Cells from Wharton Jelly; IV, Intravenous; IMV, Invasive mechanical ventilation; ARDS, Acute respiratory distress syndrome.

Although most studies focus on acute COVID-19, there is a growing interest in evaluating MSCs for persistent viral infections. A limited number of trials are investigating MSCs or their derivatives in chronic or latent infections and their associated complications, as summarized in **Table 2**, with some reporting encouraging outcomes. For instance, in HIV-positive individuals with poor immune recovery, allogeneic UC-MSC infusions increased CD4^+^ T-cell counts and enhanced HIV-specific responses without boosting viral load (246). However, these therapeutic effects appear to depend on the MSC source, as AD-MSC infusions failed to improve immune recovery in immune non-responder patients, showing no significant changes in lymphocyte subset phenotypes or in the inflammatory parameters analyzed (245).

**Table 2:** Clinical studies registered on clinicaltrials.gov that evaluate the safety and efficacy of MSCs-based therapies against persistent viral infections.

Title of the trial	MSC source	Virus	Intervention/Outcome measures	Study status or results	References/ID
Mesenchymal Stem Cells for Immune Non-responder Patients With HIV Infection	hUC-MSCs	HIV	Participants in the experimental group receive continuous antiviral therapy and MSC treatment on Day 0,30, 60 and follow up for 48 weeks. Total CD4+ T cell counts will compare with counts at baseline.	No published results. The study is currently recruiting participants.	https://clinicaltrials.gov/study/NCT05872659
HUC-MSC for Treatment of High-risk HPV Infection	hUC-MSCs	HPV	Participants in the experimental group receive 1×10^6/Kg UC-MSCs at a controlled rate of 60–80 drops/min. Adverse events will be measured on the 1^st^ day, 1^st^ week, 4^th^ week, 12^th^ week, and 36^th^ week after the infusion. HPV 24 genotypes will be tested on the 4^th^ week, 12^th^ week, and 36^th^ week.	No published results. The study is currently recruiting participants.	https://clinicaltrials.gov/study/NCT06610773
Mesenchymal Stem Cells Treatment for AIDS Patients at Late Stage	hUC-MSCs	HIV	Participants in the experimental group are randomized to receive a standard treatment administrated three times (at week 0, 2,4 after recruitment) and an enhanced treatment administrated six times (at week 0, 2,4,24,26,28 after recruitment). The number of participants with side effects and CD4+ T cells counts after MSCs transfusion will be measured after week 12, 24 and 48 and compared with baseline.	. No published results. The study is currently recruiting participants.	https://clinicaltrials.gov/study/NCT05939167
Stem Cell Study for Long COVID-19 Neurological Symptoms (COVID-19)	hUC-MSCs	SARS-CoV-2 (Long-COVID)	Participants in the experimental groups receive dose escalation using 4X10^6 cells/kg, 6X10^6 cells/kg and 8X10^6 cells/kg and 10X10^10 cells/kg (3 subjects per group). It will be determined the MTD assessed by physical exam, clinical lab measures, vital signs and subject report of adverse events. Participants will have a brain PET and MRI scan at the baseline and 6 months post-infusion visits.	No published results. The study status is not yet recruiting.	https://clinicaltrials.gov/study/NCT06156241
Mesenchymal Stem Cells Transplantation for Liver Cirrhosis Due to HCV Hepatitis	AD-MSC	HCV	Participants receive 1 million cells per kg. via peripheral vein every week for 3 times and 3 million cells per kg into the right hepatic artery for 3 times in every 2 weeks. Patients will be monitored by liver biopsies before and at 6^th^ month after the treatment, monthly biochemical and hematologic blood tests and periodic radiologic examinations.	No published results. The study status is Unknown. A previous study reported that autologous MSC transplantation via peripheral vein is safe and feasible. In two patients with non-responder hepatitis C, HCV RNA levels became negative after MSC transplantation.	(243) https://www.clinicaltrials.gov/study/NCT02705742
hUC Mesenchymal Stem Cells (19#iSCLife^®^-LC) in the Treatment of Decompensated Hepatitis B Cirrhosis.	hUC-MSCs	HBV	Participants in the experimental group receive before the 30 min of first time to inject stem cells, Intravenous methylprednisone 20mg. All patients require oral nucleoside drugs resistant hepatitis B virus treatment. Dose of stem cell therapy is 6*10^7 (30 ml). Participants are evaluated by evaluating the Model for end-stage Liver Disease score.	No published results. The study is currently recruiting participants.	https://www.clinicaltrials.gov/study/NCT03826433
Effectiveness and Safety of Mesenchymal Stem Cell Therapy in Long COVID Patients	hUC-MSCs	SARS-CoV-2 (Long-COVID)	Participants in the experimental group receive intravenous infusion of MSCs once or an additional infusion on days 35–42 if there is no significant effect at day 28. Basic physical examinations, bloodwork routine, biochemical indexes, oxygen saturation (SpO2) levels, 6-minute walk tests, HRCT scan (if necessary) will be measured at 28 days, 12 weeks, and 24 weeks after treatment completion.	No published results. The study is currently recruiting participants.	https://www.clinicaltrials.gov/study/NCT06492798
Umbilical Cord Mesenchymal Stem Cells Transplantation Combined With Plasma Exchange for Patients With Liver Failure	hUC-MSC	HBV	Participants in the experimental groups receive any of the following options: conventional therapy, conventional therapy plus hUC-MSC, conventional therapy plus PE, conventional therapy plus combined hUC-MSC and PE therapy. Survival rate after 48 weeks and clinical symptoms along with biochemical markers and liver function evaluation after 24 weeks will be measured.	UC-MSCs combined with PE treatment had good safety but cannot significantly improve the short-term prognosis of patients with HBV-ACLF as compared with the single treatment. The long-term efficacy should be further evaluated.	(244) https://www.clinicaltrials.gov/study/NCT01724398
Treatment of Long COVID Symptoms Utilizing Autologous Stem Cells Following COVID-19 Infection	AD-MSC	SARS-CoV-2 (Long-COVID)	The participants in the experimental group receive a single administration of AD-MSC (“ATCell™”). Safety will be evaluated through clinical assessments and laboratory test and the assessment of change in health status will be evaluated by using a survey with 36 item determined and compared to baseline after 1, 2, 3 and 4 weeks post administration.	No published results. The study status is Unknown.	https://www.clinicaltrials.gov/study/NCT05669261
Treatment With MSC in HIV-infected Patients With Controlled Viremia and Immunological Discordant Response	AD-MSC	HIV	Participants in the experimental group received 1 million cells/Kg MSCs at weeks 0, 4, 8, and 20. Study endpoints (incidence of adverse reactions, incidence of opportunist diseases, and changes in CD4^+^ T cell counts) were measured along a follow-up period of 24 months.	There were no significant changes in the phenotype of different immunological lymphocyte subsets, the inflammatory parameters analyzed, and cellular associated HIV-DNA in peripheral blood mononuclear cells. The findings suggest that allogeneic AD-MSC infusions are not effective to improve immune recovery in INR patients or to reduce immune activation or inflammation.	(245) https://www.clinicaltrials.gov/study/NCT02290041
ExoFlo™ Infusion for Post-Acute COVID-19 and Chronic Post-COVID-19 Syndrome	BM-MSC	SARS-CoV-2 (Long-COVID)	The participants in the experimental group will receive 10.5 x 10^8 EV. It will be measured the increased distance on six-minute walk test and the incidence of serious adverse events.	The study was withdrawn because no longer part of development plan.	https://www.clinicaltrials.gov/study/NCT05116761
Umbilical Cord Mesenchymal Stem Cells for Immune Reconstitution in HIV-infected Patients	hUC-MSC	HIV	The participants in the experimental group are randomized to receive a low or high dose of MSC at weeks 0,4,12, 24, 36 and 48 (0.5*10^6/kg or 1.5*10^6/Kg, respectively). Participants will have a physical exam and will be questioned about any medications they are taking and how they are feeling and will have blood drawn to assess CD4/CD8 cell counts and viral load at baseline and at week 4, 12, 24, 36,48,60,72,84and 96.	hUC-MSC transfusions were well tolerated and increased circulating naive and central memory CD4 T-cell counts and restored HIV-1-specific IFN-γ and IL-2 production in the INR.	(245, 246) https://www.clinicaltrials.gov/study/NCT01213186
MSC for Treatment of CMV Infection	Unspecified	CMV	Participants receive a dose of 1×10^6 cells/kg. If anticipates do not attain the complete remission standards within 14 days, a second course of the same treatment will be given. Primary and secondary outcomes were percentage of participants achieved complete remission of CMV infection and number of participants with serious and non-Serious adverse events, respectively.	No published results. The study status is Unknown	https://www.clinicaltrials.gov/study/NCT02083731
Allogeneic Bone Marrow Mesenchymal Stem Cells Transplantation in Patients With Liver Failure Caused by Hepatitis B Virus (HBV)	BM-MSC	HBV	Participants in the experimental groups receive dose escalation using 2×10^5/Kg, 1×10^6/Kg or 5×10^6/Kg once a week, 4 times (30 subjects per group). Primary outcomes measures are liver function by assessing biochemical parameters and immune status through cytokines assessment.	Treatment was safe and well tolerated. BM–MSC treatment improved clinical laboratory measurements, decreased the incidence of severe infection and had a lower mortality rate in patients with HBV-related ACLF compared with the control group.	(247) https://clinicaltrials.gov/study/NCT01322906
Efficacy and Safety of Umbilical Cord Mesenchymal Stem Cells in the Treatment of Long COVID-19	hUC-MSC	SARS-CoV-2 (Long-COVID)	Participants will receive hUC-MSCs three times with one-month interval. Six min walking distances and lung function were the primary outcome measures.	No published results. The study was withdrawn because difficult in the recruitment.	https://clinicaltrials.gov/study/NCT05719012
Study of Allogeneic Adipose-Derived Mesenchymal Stem Cells to Treat Post COVID-19 “Long Haul” Pulmonary Compromise.	AD-MSC	SARS-CoV-2 (Long-COVID)	Participants in the experimental group will receive intravenous infusions of COVI-MSC (one vial, ~18.5 million cells) on Day 0, Day 2, and Day 4. Changes in 6MWD at Day 60, in (PFTs), in oxygenation, and biomarker levels will be measured.	No published results. The study was withdrawn because was replaced by a different protocol.	https://clinicaltrials.gov/study/NCT04909892
Study of Allogeneic Adipose-Derived Mesenchymal Stem Cells to Treat Post COVID-19 “Long Haul” Pulmonary Compromise (BR)	AD-MSC	SARS-CoV-2 (Long-COVID)	Participants in the experimental group will receive 2, 4 or 6 MSC vials (approximately 15 million cells/vial) will be intravenously infused on Day 0, Day 2, or Day 4 depending on assignment to treatment group. Group A: 2 MSC vials infused on day 0 and 2 vials of placebo on day 2 and 4. Group B: 2 MSC vials infused on day 0 and 2, and 2 vials of placebo on day 4 Group C: 2 MSC vials infused on day 0 and 4, and 2 vials of placebo on day 2 Group D: 2 MSC vials infused on day 0,2, and 4. Changes in 6-Minute Walk Distance (6MWD) at Day 60, in PFT, in oxygenation, and biomarker levels.	No published results. The study was withdrawn.	https://clinicaltrials.gov/study/NCT04992247
Randomized Double-Blind Phase 2 Study of Allogeneic HB-adMSCs for the Treatment of Chronic Post-COVID-19 Syndrome (HBPCOVID02)	AD-MSC	SARS-CoV-2 (Long-COVID)	Participants in the experimental group will receive 200 million of MSC at weeks 0, 2, 6, and 10. Changes From Baseline in Visual Analog Scale of Neurological Symptoms. Changes in laboratory values, in physical examinations will be determined at baseline, week 10 and week 26.	Treatment was safe and well tolerated. Both groups showed improvement in neurological symptoms over 26 weeks, with no significant difference between MSC and placebo.	https://clinicaltrials.gov/study/NCT05126563
Long Term Follow up Mesenchymal Stem Cell Therapy for Patients Virus-related Liver Cirrhosis	BM-MSC	HCV and HBV	Participants in the experimental group will receive a single dose of 0.5 to 1 x 10^6/kg autologous BM-MSCs (Total volume: 30–50 ml).	No published results. The study is ongoing, but no recruiting.	https://clinicaltrials.gov/study/NCT05080465

hUC-MSCs, Human umbilical cord-derived mesenchymal stem cells; AD-MSCs, Adipose-derived mesenchymal stem cells; IV, Intravenous; HIV, Human immunodeficiency virus; HPV, Human papillomavirus; SARS-CoV-2, Severe acute respiratory syndrome coronavirus 2; HBV, Human hepatitis B virus; HCV, Human hepatis C virus; CMV, Cytomegalovirus; MTD, Maximum tolerated dose; PET, Positron emission tomography; MRI, Magnetic Resonance Imaging; HRCT, High-resolution computed tomography; PE, Plasma Exchange; 6MWD, 6-Minute Walk Distance; PFT, Pulmonary Function Tests; INR, immunological non-responders; ACLF, Acute-on-Chronic Liver Failure.

In the case of HBV, one study reported that BM–MSC treatment improved clinical laboratory measurements, decreased the incidence of severe infection, and lowered mortality in patients with HBV-related acute-on-chronic liver failure (ACLF) compared with the control group (247). By contrast, combining UC-MSCs with plasma exchange (PE) demonstrated good safety for patients with liver failure but did not significantly improve the short-term prognosis compared with PE alone (244). Moreover, a case report described an HBV-related ACLF patient treated with repeated PE and UC-MSCs in combination with antiviral therapy (entecavir, ETV), who survived with marked improvements in hepatic function (248). In addition, a 24-month prospective study found that UC-MSC transplantation combined with PE and ETV was safe and effective, resulting in a higher cumulative survival rate than PE treatment alone (249).

While these findings highlight the immunomodulatory potential of MSCs for chronic viral infections, their effects on associated neurocognitive disorders remain unexplored in controlled clinical settings, with a clear translational gap between preclinical findings and human application. To date, only a single study has reported results in four patients with SSPE treated with MSCs, in which no clinical benefit was observed (250). Notably, a prospective, non-randomized observational registry study is currently underway to evaluate the clinical outcomes of patients with autoimmune or post-infectious neuroinflammatory syndromes receiving MSC-based therapies. This includes post-viral encephalopathies following measles, CMV, EBV, and SARS-CoV-2, as well as early-stage panencephalitis and chronic neuroimmune syndromes such as long COVID with CNS involvement (https://clinicaltrials.gov/study/NCT07145502). It is likely that in the coming years additional data will emerge, particularly from ongoing studies evaluating MSCs in long-COVID.

### 4.3 Emerging strategies to improve MSC therapeutic efficacy and overcome barriers to translational application

The clinical translation of MSC-based therapies in viral CNS infections faces several challenges including persistent viral reservoirs, chronic inflammation, and the specialized CNS immune microenvironment. Moreover, MSC experience variable efficacy and short-term activity *in vivo* (**Figures 2A-D**) (217, 251). Therefore, strategies to precondition or engineer MSCs are critical to enhance their therapeutic robustness and reproducibility (252) Emerging technologies are being developed to enhance MSC and EV functionality, improve delivery efficiency, and ensure consistent therapeutic outcomes (**Figures 2E-H**) (176, 198, 253).

Preconditioning approaches aim to “prime” MSCs by exposing them to specific environmental cues before transplantation, thereby activating protective and therapeutic pathways (254, 255). For instance, hypoxic preconditioning (1–5% O_2_) mimics the low oxygen tension of the native MSC niche, stabilizing hypoxia-inducible factors (HIFs) that upregulate glycolytic enzymes and promote a shift toward aerobic glycolysis (256, 257). This metabolic reprogramming enhances MSC survival, mitochondrial fitness, and resistance to oxidative stress, while boosting the secretion of trophic and angiogenic factors such as VEGF, BDNF, and HGF, thereby increasing regenerative and neuroprotective efficacy (256, 257). Similarly, inflammatory cytokine preconditioning using factors such as IFN-γ, TNF-α, or IL-1β upregulates key immunoregulatory molecules including IDO, TSG-6, and PD-L1, improving the MSC capacity to dampen immune responses, which could potentially inhibit viral-associated inflammation (**Figure 2E**) (258, 259).

In parallel, genetic engineering strategies are increasingly employed to overcome inherent limitations of MSCs (198, 260). Using viral vectors or CRISPR/Cas9-based systems, MSCs can be modified to overexpress therapeutic genes such as CXCR4 (to improved homing), IL-10, BDNF, or IFN-β, thereby enhancing their immunomodulatory, antiviral, and neurotrophic potential (170, 173, 261, 262). On the other hand, bioengineered EVs have been conjugated with heparin for enhancing brain targeting and MSC preconditioning with interferon γ or viral antigens for boosting IFN-β secretion while maintaining immunomodulatory capacity (**Figure 2F**) (263, 264).

Hydrogel scaffolds that sustain EV release may support long-term miRNA delivery, maintaining therapeutic thresholds in chronic infections (**Figure 2G**) (265, 266). Metabolic modulation represents another frontier, where exposure to agents that shift metabolism towards glycolysis by increasing the expression of certain glucose transporters can enhance MSC resistance to stress and alter the composition of their secretome toward a more anti-inflammatory and neuroprotective profile (**Figure 2H**) (267). Additionally, chemical preconditioning using small molecules (e.g., valproic acid, resveratrol) has also been explored to boost anti-apoptotic and antioxidant properties (268, 269).

Collectively, these bioengineering strategies, including hypoxic and inflammatory preconditioning, metabolic reprogramming, chemical priming, and genetic modification, are essential tools to optimize MSC function, especially under the hostile conditions of viral CNS infections. Their implementation could significantly improve the reproducibility, robustness, and clinical impact of MSC-based therapies.

## 5 Concluding remarks

Viral infections of the CNS represent a significant global health burden due to their capacity to induce persistent neuroinflammation and long-term neurological sequelae. Given the current state of evidence, future research should prioritize the design of well-powered randomized controlled trials targeting viral CNS infections, rather than extrapolating from systemic viral infections or non-viral neuroinflammatory disorders. Standardization of MSC manufacturing, dose regimens, and delivery routes will be essential to reduce variability and ensure reproducibility (270). Long-term follow-up studies will be critical to assess the durability of therapeutic benefits and to detect any delayed adverse events that may arise from MSC transplantation. Moreover, mechanistic studies in human subjects are needed to determine the precise contributions of MSCs to viral clearance, host immune responses modulation, and repair processes within the CNS (271). This knowledge will be pivotal for refining patient selection criteria and optimizing treatment regimens tailored to specific viral etiologies (194, 272). Emerging strategies, including artificial intelligence-driven secretome optimization and 3D bioprinting of MSC-laden scaffolds, aim to standardize production and enhance therapeutic consistency (273). Furthermore, combining MSC-EVs with nanotechnology-based delivery systems could improve CNS targeting, offering a translatable solution for viral infections like HAND or HSE. Combining MSC-based strategies with antiviral or immunomodulatory agents may offer synergistic benefits; however, this remains to be fully evaluated. While the therapeutic potential of MSC-based therapies is substantial, it is essential to approach this field with scientific rigor, and an interdisciplinary approach will be crucial to move MSC-based interventions from bench to bedside in the treatment of virus-induced neuroinflammatory diseases.
